# Therapeutic Potential of Adipose-Derived Regenerative Cells for Ischemic Diseases

**DOI:** 10.3390/cells14050343

**Published:** 2025-02-27

**Authors:** Yiyang Che, Yuuki Shimizu, Toyoaki Murohara

**Affiliations:** Department of Cardiology, Nagoya University Graduate School of Medicine, Nagoya 466-8550, Japan

**Keywords:** ADRC therapy, therapeutic angiogenesis, paracrine, extra vesicle, cell-to-cell communication

## Abstract

Adipose-derived regenerative cells (ADRCs) are one of the most promising cell sources that possess significant therapeutic effects. They have now become a main source of cell therapy for the treatment of ischemic diseases due to their easy accessibility, expansion, and differentiation. Additionally, ADRCs can release multiple paracrine factors and extracellular vesicles that contribute to tissue regeneration by promoting angiogenesis, regulating inflammation, alleviating apoptosis, and inhibiting fibrosis. However, ADRCs still have some limitations to realize their full therapeutic potential. To address these issues, protective mechanistic studies and bioengineering studies have been carried out. This review focused on the recently studied mechanisms, such as paracrine factors, cell fusion, and mitochondrial transfer, involving the therapeutic potential of ADRCs in ischemic diseases and discussed some modification techniques of ADRCs.

## 1. Introduction

A decrease in the blood flow, known as ischemia, results in a lack of the oxygen and nutrient supply required for cellular function and is mainly caused by a blockage in the arterial blood flow in a tissue, organ, or extremity [1]. Ischemic diseases include cerebrovascular diseases, ischemic heart diseases, peripheral artery diseases, and ischemic kidney diseases, whose patients are at significant risk for cardiovascular events, amputation, and mortality.

Over the decades, therapeutic angiogenesis has been proposed to stimulate the regeneration/reconstruction of vascular networks in ischemic tissues and enhance blood flow by gene therapy (and cytokines) or cell therapy. Due to the wide range of the therapeutic potential of stem cells, cell therapy has become an attractive approach in regenerative medicine. Adipose-derived regenerative cells (ADRCs) are especially promising among stem/progenitor cells because of their great accessibility, low immunogenicity, mass volume, and less invasive collection. As a result, ADRCs have become an attractive cell source for clinical application in therapeutic angiogenesis for ischemic diseases.

This review explores the therapeutic effects and potential mechanisms of ADRCs in ischemic diseases, summarizes recent bioengineering methods of ADRCs, and presents new perspectives on ADRC therapy.

## 2. Characteristics of Adipose-Derived Regenerative Cells

ADRCs are a type of somatic stem cell that can be easily separated from the adipose tissue while carrying out regenerative capabilities. They were initially identified by Zuk et al. in 2001 [2] as a population in the subcutaneous adipose tissue that was able to adhere to plastic, self-renew, and differentiate into multiple lineages such as neurons, hepatocytes, endothelial cells, osteoblasts, adipocytes, myocytes, and chondrocytes [3,4], similar to bone marrow-derived mesenchymal stem cells. The differentiation of ADRCs in vitro can be induced by a selective medium with specific factors [5]. Although ADRCs are difficult to differentiate into vascular endothelial cells, they can successfully differentiate into vascular endothelial cells and interconnect with the vascular network when they are co-cultured with human umbilical vein endothelial cells (HUVEC) in an EGM-2 medium supplemented with growth factors [4]. In addition, most clinical applications employ appropriate biomaterial scaffolds capable of supporting ADRC adhesion, proliferation, and differentiation, hence providing an optimum environment for cell survival. The scaffolds supporting ADRCs should be bioactive with low immunogenicity and good mechanical integration [6]. A study of an ischemia model has revealed that a three-dimensional (3D) culture based on ADRC adhesion to a substrate enhances the endothelial differentiation of ADRCs [7]. Furthermore, compared to bone marrow-derived cells, ADRCs can be collected in large quantities from subcutaneous regions more easily, and they are relatively less invasive to collect while having nearly the same potential. Studies have also shown that ADRCs exhibit lower immunogenicity, greater proliferative ability, and more immunosuppressive potential than bone marrow-derived mesenchymal stem cells [8]. Therefore, ADRCs brought up new possibilities for regenerative medicine.

**Figure 1:**
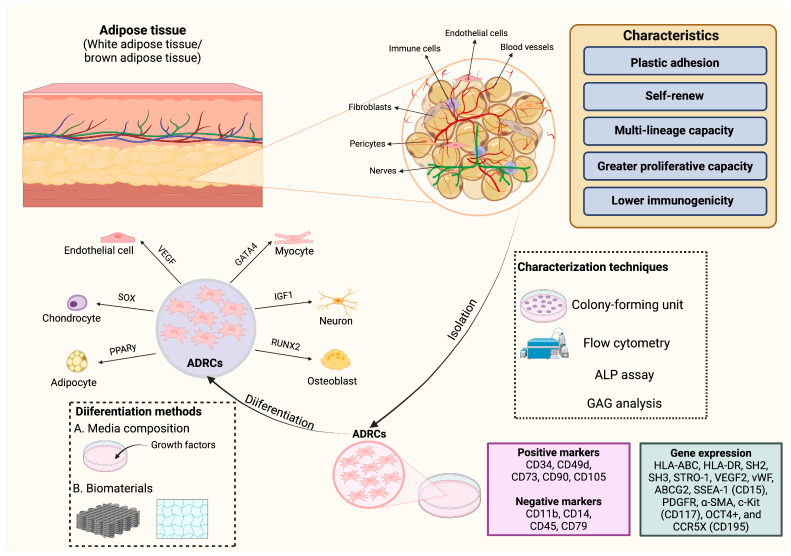
Characteristics of ADRCs. This figure was created with BioRender (version 1.0.0.3).

In terms of the stemness of ADRCs, colony-forming unit (CFU), flow cytometry, and differentiation tests such as ALP assay, GAG analysis, and oil red staining [9] were used to evaluate the proliferation rate, self-renewal ability, and multi-lineage ability of ADRCs (Figure 1). The ability of stem cells to form colonies indicates their potency and proliferation, and it is calculated by culturing the cells in a medium for 10–14 days and then seeing and counting the colonies with crystal violet dye [10]. Flow cytometry is also used to characterize cells based on the expression of surface markers [11]. The surface markers of ADRCs are typically identified by treating subcultured cells with primary monoclonal and secondary antibodies tagged with dyes [12]. In addition, ADRCs show positive expression of the mesenchymal cell surface markers like CD73, CD90, and CD105, but negative expression of CD11b, CD14, CD45, and CD79 [13,14]. Also, ADRCs can express CD34 and CD49d, whereas bone marrow-derived stem cells cannot [15]. Other expression genes, including HLA-ABC, HLA-DR, SH2, SH3, STRO-1, VEGF2, vWF, ABCG2, SSEA-1 (CD15), PDGFR, α-SMA, c-Kit (CD117), OCT4+, and CCR5X (CD195), have also been reported [16]. Therefore, for a better application in regenerative medicine, more specific markers for ADRC identification and standard identification protocols need to be further investigated.

## 3. ADRC Therapies and Ischemic Diseases

### 3.1. ADRC Therapies

**Figure 2:**
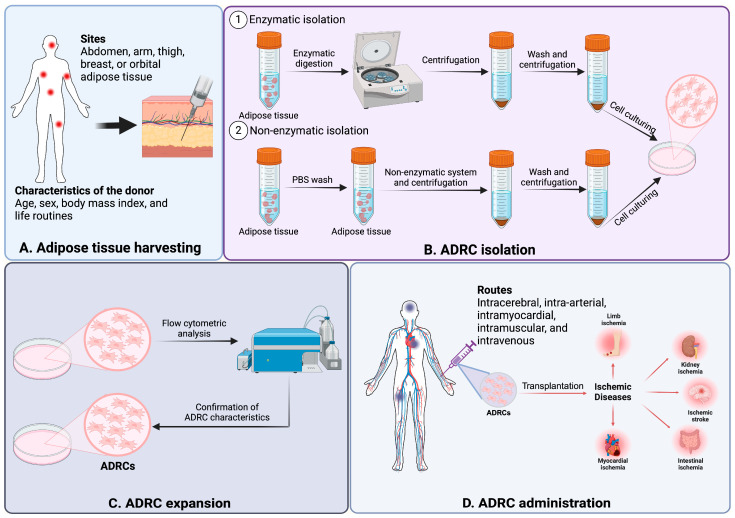
ADRC collection and transplantation. This figure was created with BioRender.

Due to the great advantages of ADRCs, they have been widely studied to treat ischemic diseases by delivering them into the ischemic tissue in regenerative medicine, and they have become an outstanding candidate in stem cell-based therapy for ischemic diseases. Studies and some clinical trials have confirmed the high therapeutic potential of ADRCs, with properties such as angiogenesis, neurogenesis, DNA damage repair, anti-inflammatory, anti-apoptosis, anti-ferroptosis, antioxidant stress, and anti-fibrosis against numbers of ischemic diseases [17]. Regarding the consistency, viability, and accessibility of ADRC isolation, they are affected by numerous factors, and it is important to understand and standardize these factors. Therefore, for a better understanding of the clinical application of ADRC therapy, the sources and donors of ADRCs, the techniques of ADRC isolation, the administration methods, and the doses of ADRCs are summarized in Figure 2.

#### 3.1.1. Source

The two primary types of adipose tissue are white adipose tissue and brown adipose tissue. ADRCs from brown adipose tissue exhibit distinct characteristics compared to those derived from white adipose tissue and are more prone to skeletal myogenic differentiation [18]. In clinical application, subcutaneous adipose tissue is mostly used for the isolation of ADRCs because of their abundant amount compared with visceral fat. ADRCs isolated from separate subcutaneous adipose tissue sites (such as the abdomen, arm, or thigh) express the same CD surface markers and exhibit similar capability for multilineage differentiation [19]. However, they show different functions in proliferation rates, therapeutic potential (such as anti-apoptosis), and paracrine factors [20,21]. Maddox et al. tested mesenchymal stem cells harvested from breast and abdominal adipose tissues for osteogenic and adipogenic differentiation. The results show that the SSEA-4 antigen appears to mark a subset of mesenchymal stem cells with a higher potential for differentiation into osteogenic and adipogenic cell lineages [22]. Furthermore, Nepali et al. showed that orbital ADRCs have a larger ability for adipogenic and osteogenic differentiation but a lower one for chondrogenesis when compared to abdominal ADRCs [23]. In addition, Ishiuchi et al. demonstrated that ADRCs from the superficial layers had higher proliferation and adipogenic differentiation abilities than ADRCs from the deep layers, despite having similar morphology, cell surface markers, senescence markers, and expression of coagulation and anticoagulant factors [24]. Regarding the angiogenesis ability of ADRCs from different sources, characterization and modification techniques can be used to identify and improve the therapeutic angiogenesis of ADRCs. In spite of ADRCs from different depots displaying distinct characteristics, they have still been effectively utilized for clinical research.

#### 3.1.2. Donor

Recent studies have shown that the quantity and quality of ADRCs can be affected by the health status and characteristics of the donor, such as donor age, sex, body mass index, and life routines. ADRCs extracted from diabetes or obesity donors showed impaired proliferative activity and secretion of angiogenic factors and expressed a proinflammatory phenotype [25,26]. In addition, donors of different ages showed different cytokine expression levels, which could also lead to alterations in the immunomodulatory and angiogenesis ability of ADRCs [27]. However, some studies also revealed that donor age had a limited effect on ADRC proliferation and yield [28]. It has also been reported that gender differences can also influence ADRC functionality in differentiation, paracrine mechanisms, and inflammation [29,30]. As for lifestyle habits, plenty of studies demonstrated that alcohol and tobacco can decrease the therapeutic potential of ADRCs [31,32], whereas moderate exercise can enhance the therapeutic effects of ADRCs [33].

#### 3.1.3. Techniques

It is well known that the adjustment of ADRC harvesting and processing techniques can influence the expected clinical outcome of ADRC therapy, and the isolation methods have become more appropriate for clinical use after years of developing and optimizing. The traditional method first introduced by Zuk et al. is the enzymatic isolation of ADRCs, which relies on collagenase digestion [2]. This has been widely applied in clinical settings after modification. In addition, non-enzymatic systems such as the Body-Jet^®^ liposuction system and the Q-graft^®^ system produced by Human Med AG (Schwerin, Germany) have also been proposed on the basis of centrifugation force, pressure, filtration, and washing for a safer and more effective system to purify the adipose tissue [34]. Continuous studies on isolation techniques of ADRCs should be further conducted for more effective applications in ADRC therapies.

#### 3.1.4. Doses and Administration

In clinical trials of the ADRC therapy application, ADRCs can be administrated through several routes, such as intracerebral, intra-arterial, intramyocardial, intramuscular, and intravenous (Table 1). In terms of the efficiency and toxicity of the ADRC therapy, the delivery dosage should also be considered for clinical application. Additionally, the immunomodulatory action of ADRCs and the outcomes of ADRC therapy can also be impacted by the local circumstances of administration. Therefore, prior to ADRC application, the inflammatory state in ischemic diseases should also be taken into account in addition to ADRC sources, isolation techniques, and doses.

### 3.2. Therapeutic Angiogenesis in Ischemic Diseases

Clinical trials have demonstrated the ADRCs’ efficacy and safety. The protective effects of ADRC against ischemia have been reported in ischemia of multiple organs, such as in ischemic heart, stroke, ischemic kidney disease, and intestinal ischemia (Table 1 and Figure 2). These are thought to be due to stimulating angiogenesis at the capillary level and/or the development of collateral blood vessels, thereby restoring ischemia. Since their viability is extremely low, it is assumed that the primary mechanism of the therapeutic effect is not a direct action (i.e., ADRCs differentiate into endothelial cells and are adopted into blood structures) but an indirect action, such as paracrine action, that contributes to the promotion of reparative angiogenesis [48]. We have conducted therapeutic angiogenesis with ADRCs for patients with chronic limb-threatening ischemia (CLTI) [49]. In those studies, we have experienced cases in which the therapeutic effect was much greater than we expected [44,45]. Therefore, from our clinical experience, we believe that there may be other mechanisms of action of ADRC besides the one we have postulated.

### 3.3. Other Therapeutic Actions

**Figure 3:**
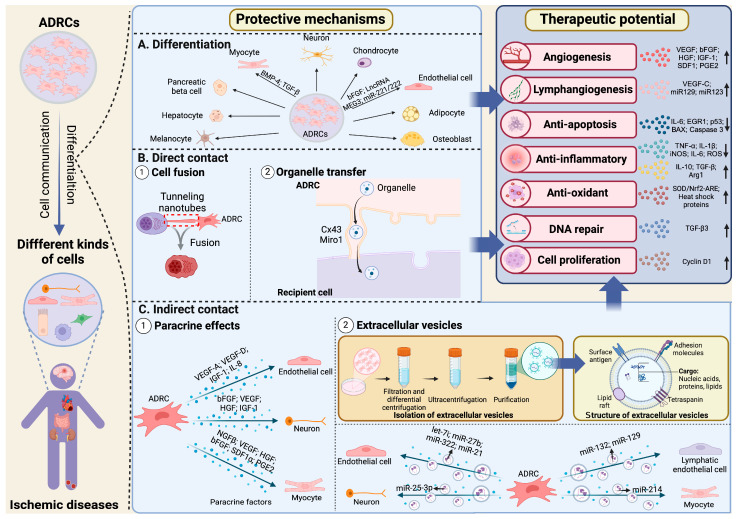
Therapeutic potential and molecular mechanisms of ADRC therapy in ischemic diseases. This figure was created with BioRender.

Wound bed preparation (WBP) has been proposed to evaluate ischemic tissue, and it is clinically important to manage it in a multidisciplinary way as well [50]. The concept is to eliminate treatment resistance factors against restoring tissue repair and blood flow by improving a reparative angiogenesis environment. To this end, it has been experimentally reported that ADRCs contribute to enhancing angiogenesis by modulating local inflammation. We demonstrated that ADRCs release PGE2 and modulate the polarity of local macrophages in ischemic tissues [51], resulting in promoting angiogenesis and effective blood perfusion recovery. We showed that ADRC can indirectly promote angiogenesis and ameliorate excessive local inflammation and tissue edema by enhancing lymphangiogenesis in ischemic tissues [52]. Thus, ADRC can promote not only angiogenesis itself but also lymphangiogenesis to maintain the regenerative environment in a hindlimb ischemia murine model. In addition, ADRC can have an anti-apoptotic action [53,54], an antioxidant action [55,56], and a DNA repair action [57,58,59], which are all possible mechanisms for protective action in damaged tissues (Figure 3).

## 4. Molecular Mechanisms of the Protective Effect by ADRCs

With their ability to self-renew and differentiate into several cell phenotypes, ADRCs provide promising treatment options for ischemic diseases. One of the potential mechanisms for the ADRC-mediated pro-angiogenic advantages is their ability to differentiate into other cells. However, it has been recently shown that the therapeutic potential of transplanting ADRCs into host tissues may not be solely due to cell replacement and differentiation but also to their indirect communication with other cells through paracrine effects and extracellular vesicle secretion [60]. ADRCs can release a variety of biological molecules, including growth factors, cytokines, and microvesicles, which contribute to their immunomodulatory, angiogenic, antiapoptotic, DNA repair, anti-fibrosis, and antioxidative effects. Moreover, ADRCs can also induce angiogenesis, suppress apoptosis, boost cell proliferation, and suppress inflammation and the immune response through direct cell contact via gap junctions (GJs), tunneling nanotubes (TNTs), and cell fusion [61] (Figure 3).

### 4.1. Differentiation

ADRCs can differentiate into a range of cell lineages and promote tissue regeneration in ischemic diseases. It has been demonstrated that ADRCs can differentiate into endothelial cells, pericytes, smooth muscular cells, fibroblasts, and immune cells with therapeutic effects, and those kinds of differentiation can be regulated by microenvironment factors like inflammation [62]. Changes in gene function during ADRC differentiation lead to the expression of genes that provide a terminally differentiated phenotype. Certain elemental families or processes, such as cell-to-cell and cell–matrix interactions, strongly control the differentiation process. There is mounting evidence that the extracellular matrix plays a part in controlling cell phenotype and differentiation by controlling cell shape and interacting directly with cell surface receptors [63]. Additional methods of causing ADRCs to differentiate into multiple lineages, including the use of pharmacological, physiological, or genetic inducers, have also been investigated, and they will be outlined in the following paragraphs.

Following the first discovery of ADRCs’ endothelial differentiation capability in 2004 [63], there have been several encouraging studies demonstrating ADRCs’ ability to differentiate into endothelial cells in vitro. According to Konno et al. [64], bFGF is a powerful inducer of endothelial cell development. The absence of bFGF significantly reduced ADRCs’ capacity to absorb Ac-LDL and decreased the expression of EC markers, including CD31, VE-Cadherin, vWF, VEGFR1, and eNOS. V. Suresh and J. L. West demonstrated how the ability of the ADRC population to develop into an endothelial cell lineage is influenced by 3D culture and cell density [65]. In addition, it has been proved by Arderiu et al. [66] that ADRCs differentiate toward endothelial cells in response to bFGF produced by endothelial cells via miR-145-regulated ETS1 expression. By targeting miR-145-5p/KLF4, LncRNA MEG3 induced endothelial differentiation of ADRCs, which might bring new insights into the process underlying the endothelial differentiation of ADRCs [67]. It has also been shown that ADRCs have the ability to differentiate into the endothelium lineage in vivo. After injecting the cells intravenously, Moon et al. [68] demonstrated the integration of ADRCs into the vasculature of the mouse ischemic hindlimb and an increased capillary density, confirming their ability to differentiate into endothelial cells in vivo. It has also been demonstrated that miR-221/222 is crucial for ADRC endothelial differentiation and offers a unique method of regulating ischemic tissue regeneration and vascular development, and angiogenesis was promoted when those ADRCs were injected into diabetic mice’s ischemic hindlimb [69].

In addition to endothelial cells, ADRCs can also differentiate into smooth muscle cells. The effective differentiation of ADRCs into smooth muscle cells was initially documented by Rodríguez et al. in 2006 [70]. Recently, Salem et al. [71] showed the ability of ADRCs to develop into smooth muscle cells following stimulation, and ADRCs displayed distinctive leiomyogenic markers at the genetic and protein levels and presented typical smooth muscle morphology. Additionally, Kim et al. [72] showed that implanted ADRCs repaired damaged smooth muscle at the site of injury, suggesting that the stem cell destiny matches that of the damaged area’s smooth muscle cells. Moreover, Yogi et al. [73] indicated that ADRCs can differentiate into vascular smooth muscle cells after 4 days of being exposed to BMP-4 and TGF-β and have the capacity to contract in response to a depolarizing substance in addition to their molecular markers. In an in vivo myocardial infarction model, the results of the intracoronary injection of autologous ADRCs demonstrated that ADRCs can develop into vascular smooth muscle cells and endothelial cells, thus enhancing cardiac function and inhibiting unfavorable ventricular remodeling by promoting angiogenesis in the infarct border zone [74].

### 4.2. Indirect Contact

By releasing large amounts of bioactive molecules such as angiogenic growth factors, ADRCs, early after aspirated fat transplantation, may promote the growth of new vessels from the recipient region, cellular migration, proliferation, and autocrine signaling. The indirect contact between ADRCs and host cells is mainly through those bioactive molecules, including growth factors, cytosolic components, extracellular vesicles, pro- and anti-inflammatory cytokines, and other substances. Additionally, microenvironments can also induce a variety of factors, such as vascular endothelial growth factor (VEGF), basic fibroblast growth factor (bFGF), and hepatocyte growth factor (HGF), released by ADRCs [75,76]. In the following section, we mainly discuss the paracrine effects and extracellular vesicles secreted by ADRCs.

#### 4.2.1. Paracrine Effects

The production of multiple paracrine growth factors is considered to be the main mechanism contributing to angiogenesis and other effects following the administration of ADRCs. According to Hsiao et al. [77], VEGF-A and VEGF-D are two of the main growth factors secreted by ADRCs that promote endothelial tubulogenesis. They also found that insulin-like growth factor-1 (IGF-1), vascular endothelial growth factor-D (VEGF-D), and interleukin-8 (IL-8) are expressed at higher levels in ADRCs than in other stem cells, which also demonstrates the advantage of ADRC therapy in regenerative medicine.

A lack of blood flow to the brain is the cause of an ischemic stroke. As a result, enhancing angiogenesis in the ischemic region may be one of the key therapeutic goals for ischemic stroke treatment. Increasing bFGF and VEGF production in the brain, ADRC therapy has been shown to contribute to the revascularization of cerebral ischemia in rats [78]. According to several reports, ADRCs may also secrete a number of trophic factors following post-stroke administration, such as VEGF, HGF [79], and IGF-1 [80]. These factors may contribute to a decrease in apoptosis resulting from brain injury and an improvement in the natural repair response.

Mechanistic research on the therapeutic effects of ADRCs has demonstrated that the therapeutic angiogenesis of ADRCs is mostly promoted by paracrine secretion. In an animal myocardial infarction model transplanted with ADRCs, the heart function and inhibition of myocardial remodeling are achieved through the release of pro-angiogenic factors such as stromal cell-derived factor-1α, nerve growth factor beta, VEGF, HGF, and bFGF [81]. It has also been demonstrated that ADRC transplantation in a limb ischemia model results in a more rapid recovery of blood flow following ischemic muscle damage and increases capillary density through the paracrine release of angiogenic molecules, including VEGFs [75,82], HGF [82], SDF1 [83] , and PGE2 [51].

#### 4.2.2. Extracellular Vesicles (EVs)

Apart from paracrine factors, extracellular vesicles (EVs) released by ADRCs have been recognized as a new mechanism of indirect cell communication. These studies showed that EVs released by ADRCs have the ability to directly stimulate angiogenesis and promote other protective effects on the damaged tissue.

By secreting EVs, ADRCs promote angiogenesis and neuroprotection in preclinical stroke models. Through released EVs containing miR-25-3p, Kuang et al. showed that ADRCs promote neuroprotection by improving the autophagic flux [84]. Currently, there is an ongoing trial (NCT06138210) for the treatment of acute ischemic stroke using EVs, and the results from this trial will not only provide insights into the safety and efficacy of EV treatments in stroke patients but will also pave the path for future clinical studies adapted to specific stroke subtypes and phases.

After the implantation of EVs from rat ADRCs or miR-126-overexpressing ADRCs under hypoxic circumstances in an in vitro model of cardiac infarction, rat endothelial progenitor cells migrated and formed tube-like structures more quickly, and this showed that ADRC EVs promote angiogenesis and have a stronger proangiogenic impact in hypoxic environments [85]. Additionally, we demonstrated that ADRCs can express a number of miRNAs (such as miR-7a, miR-19a, miR-221, and miR-222) using quantitative RT-PCR, with miR-214 being the most prevalent, and the anti-apoptotic effects of ADRC therapy on cardiomyocytes in vitro and in vivo were impaired by miR-214 silencing in ADRCs [86]. An ongoing trial (NCT05669144) is currently examining the combined transplantation of mitochondria and the administration of mesenchymal stem cell-derived EVs in patients with myocardial ischemia. Additional longitudinal clinical studies are required to validate the safety and efficacy of exosome therapy.

EVs derived from ADRCs have been shown to stimulate angiogenesis in hind limb ischemia. We identified that let-7i, miR-27b, miR-322, and miR-21 in exosomes produced from ADRCs can promote angiogenesis and enhance blood perfusion in a mouse model of hind limb ischemia [87]. Additionally, in limb ischemia models, it has been shown that EVs produced from ADRCs have pro-angiogenic and skeletal muscle protective characteristics related to their NRG1/mRNA cargo, hence promoting therapeutic angiogenesis and muscle protection [88].

It has also been discovered that ADRC EVs promote lymphangiogenesis. In their study of ADRC EVs’ function in lymph tissue growth via VEGF-C, Wang et al. discovered that ADRC EVs co-cultured with recombinant VEGF-C enhanced lymphatic endothelial cell (LEC) migration, proliferation, and tube formation more than EVs cultured without VEGF-C, and this effect is related to the elevated levels of miR-132 [89]. According to Yang et al. [90], hypoxia ADRC-isolated EVs may be a useful target for the treatment of disorders linked to lymphatic remodeling because they demonstrated that these EVs significantly promote the proliferation, migration, and tube formation of LECs, and this effect is mediated by miR-129/HMGB1/AKT signaling.

### 4.3. Direct Contact

Although it is primarily believed that the regenerative abilities of ADRCs happen through paracrine effects such as the production of trophic factors, ADRCs can also exert their effects through cell–cell direct contact (juxtacrine interactions). The activation of juxtacrine receptors like Notch and the stability of growing sprouts by direct cell–cell interactions are examples of believable juxtacrine mechanisms of action [91]. Through these processes, ADRCs can modify the differentiation status, survival, and synthesis of the extracellular matrix (ECM), matrix metalloproteinases (MMPs), and tissue inhibitors of metalloproteinases (TIMPs) by fibroblasts and myofibroblasts, and ECM, MMPs, and TIMPs are also produced by ADRCs, which aid in the regeneration process [92].

#### 4.3.1. Cell Fusion

Permanent cell fusion between stem and resident cardiomyocytes can contribute to the restoration of damaged myocardium by producing new cardiomyocytes in response to impairment. It has previously been shown that stem cells may fuse with cardiomyocytes through either permanent or partial cell fusion processes [93]. Co-culturing stem cells and cardiomyocytes in vitro to simulate the cardiac environment revealed that hybrid cells can be produced by a mechanism other than permanent cell fusion. This mechanism can involve partial cell fusion through temporary direct cell-to-cell communication and intercellular exchange of different substances [94]. The basis for this temporary cell fusion process, which was found in 2004, is the creation of membrane thin channels, known as TNTs, which enable membrane continuity between linked cells [95].

Acquistapace et al. [96] found that heterologous cell fusion between cardiomyocytes and ADRCs can induce cardiomyocyte reprogramming back to a progenitor-like state. In addition to having a mouse genotype, the resultant hybrid cells displayed early cardiac commitment and proliferation markers, such as GATA-4, myocyte enhancer factor 2C, Nkx2.5, and Ki67. Also, they demonstrated that partial rather than permanent cell fusing was the best approach for producing cardiac hybrid cells. Metzele et al. [97] also demonstrated that ADRCs can fuse with neonatal rat cardiomyocytes in vitro, and most of the fused cells displayed cardiomyocyte-like morphology and spontaneous rhythmic contraction. After fusing with rat cardiomyocytes, ADRCs show both stem cell (proliferation) and cardiomyocyte (action potential and spontaneous rhythmic beating) characteristics. In the cellular fusion between ADRCs and the mouse myoblast C2C12 cell system, Eom et al. [98] demonstrated that ADRCs expressing dystrophin and myosin heavy chain (MyHC) contribute to generating myotubes during co-cultivation with mouse myoblast C2C12 cells.

#### 4.3.2. Organelle Transfer

Other than cell communication mechanisms such as cell fusion and EV trafficking, organelle transfer through TNTs or GJs is also one of the mechanisms underlying the therapeutic effects of ADRCs. One of the important regulators in controlling the transport of mitochondria is the mitochondrial motor protein Rho-GTPase 1 (Miro1). In addition to Miro1, Zhang et al. [99] discovered that TNF-α promotes mitochondrial transfer to cardiomyocytes by inducing TNT production in stem cells through the TNF-α/NF-κB/TNFαIP2 signaling pathway. ROS signals have been shown to promote the synthesis of TNTs [100]. Additionally, connexin 43 (CX43) controls the transfer of mitochondria from stem cells through the creation of TNTs [101]. Furthermore, GJ channels that contain CX43 are essential for facilitating the transfer of mitochondria [102]. Additionally, in vitro investigations discovered that this spontaneous mitochondrial transfer across cells may also be bidirectional, and this phenomenon is important to look into its possible function in promoting tissue regeneration [103,104].

Intercellular mitochondrial transfer mediated by ADRCs has recently been demonstrated to be a non-negligible biological event in the central nervous system and is believed to play important roles in the recovery from ischemic and hemorrhagic damage rescue and neurodegeneration. In the model of damaged neural stem cells caused by cisplatin, Boukelmoune et al. [105] demonstrated that stem cells can transfer mitochondria to neural stem cells in order to protect neural stem cells from the neurotoxic effects of cisplatin treatment. Additionally, the overexpression of Miro1 in stem cells enhanced mitochondrial transfer and further enhanced the survival of neural stem cells treated with cisplatin. Thus, they came to the conclusion that one way stem cells achieve their therapeutic regenerative benefits following cisplatin therapy is by mitochondrial transfer.

ADRCs can also exhibit the therapeutic potential to rescue ischemia-exposed cardiomyocytes from cell death by the mitochondrial transfer mechanism. In the investigation of mitochondrial transfer in co-cultures of rat cardiomyocytes and human ADRCs kept in hypoxic conditions, Mori et al. [106] found that mitochondrial transfer in vitro necessitated the development of cell-to-cell contacts and synergistically enhanced energy metabolism. They also found donor mitochondrial DNA in the recipient myocardium alongside a notable increase in cardiac function.

## 5. Modifications of ADRCs

**Figure 4:**
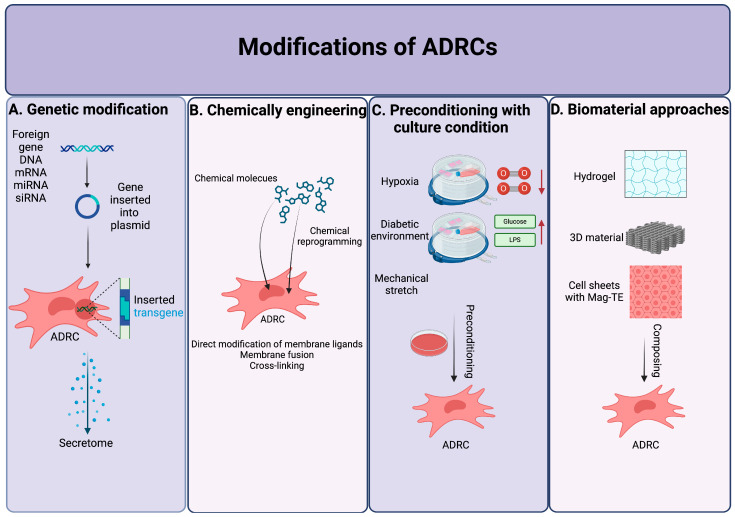
Modification methods for improving the functions of ADRCs. This figure was created with BioRender.

The therapeutic efficacy of ADRCs is still debatable despite its exceptional preclinical advantages, as clinical research using ADRCs has often had limited results. In order to overcome this obstacle, researchers have developed a number of modification techniques to produce highly functioning ADRCs. These techniques include genetically modifying ADRCs, preconditioning ADRCs with different stimulants, such as inflammatory chemicals, and altering culture conditions, such as hypoxic circumstances, to modify ADRCs (Figure 4).

### 5.1. Genetic Modification

Pretreatments including genetic alteration may improve the therapeutic angiogenic effects of ADRCs. To fully use their potential, ADRCs are genetically modified by precisely and carefully controlling the expression of certain gene sequences using a variety of techniques.

In order to create v-myc-expressing ADRCs, Song et al. [107] used a lentiviral gene transfer technique to introduce v-myc into ADRCs. They discovered that v-myc ADRCs release more VEGF and have a higher ability for migration and proliferation than normal ADRCs. After that, they examined how the culture media from genetically engineered ADRCs treated an animal model of cutaneous wounds and discovered that it promoted angiogenesis, which accelerated wound healing. After EV-donor ADRCs were preconditioned with endothelial differentiation medium (EDM), Kang et al. [108] found that the level of miR-31 in EVs was significantly increased. Their subsequent research showed that EVs from ADRCs, especially those that were preconditioned with EDM, stimulate angiogenesis, and that the delivery of miR-31 may have a proangiogenic effect. In order to create modified ADRCs with much higher levels of VEGF secretion (VEGF-ADRCs), Shevchenko et al. [109] transduced ADRCs using a recombinant adenoassociated virus expressing VEGF165. In addition, transplantation of VEGF-ADRCs in a mouse ischemia hindlimb model promoted angiogenesis and improved reperfusion and revascularization. We demonstrated that ADRCs induced by six factors (Baf60c, Gata4, Gata6, Klf15, Mef2a, and Myocd) express several cardiac genes and promote cardiac differentiation [110]. Also, the in vivo injection of those genetically modified ADRCs into acute myocardial infarcted tissues improved the infarct scar area, fractional shortening, and survival rate. Thus, genetic modifications in ADRC therapy can be used to develop a promising approach to the treatment of ischemic diseases.

### 5.2. Chemically Engineering

One of the most effective ways to enhance cell activity and therapeutic potential is to precondition ADRCs with certain biological and biochemical stimuli in vitro. By causing phenotypic changes, genetic modification, and signaling pathway activation, pre-conditioning with chemical molecules can change ADRC functions, including anti-apoptosis, differentiation, regeneration, and migration. It has been widely studied that preconditioning with inflammatory cytokines or mediators improves the immunomodulatory qualities and therapeutic potential of ADRCs.

As reported by Liu et al. [111], pretreatment with curcumin, a strong antioxidant with anti-inflammatory qualities that is obtained from the spice turmeric, increased neovascularization and decreased cardiac apoptosis, which enhanced the survival of the ADRCs that were transplanted and improved myocardial regeneration. In addition, Tao et al. [112] observed that Metformin can enhance the angiogenic capacity and autophagy of ADRCs, and Metformin-induced autophagy was associated with increased VEGF-A production and release, which contributes to promoting the angiogenesis and therapeutic efficacy of ADRCs. Wang et al. [113] showed that the LXR agonist T0901317 increases the survival and retention of intramyocardially injected ADRCs partially through the TLR4/NF-κB and Keap-1/Nrf-2 signaling pathways. In addition, a combination treatment with the LXR agonist T0901317 and ADRCs attenuates fibrosis, inhibits host cardiomyocyte death, and enhances cardiac function, producing growth factors (VEGF, bFGF) and decreasing inflammatory cytokines (IL-6) in the infarct myocardium.

### 5.3. Preconditioning with Culture Condition

The integrity of the stem cell population has been demonstrated to depend on the stem cell niche. Considering quality and quantity concerns, it is crucial to create niche-like culture conditions in order to generate clinical-grade ADRC lines. Therefore, a number of studies have found that altering the standard culture methodology may increase the therapeutic effectiveness of ADRCs.

Barone et al. [114] found that preconditioning with hypoxic condition enhances the pro-angiogenic actions of ADRC-conditioned media by producing a secretome rich in pro-angiogenic soluble factors, the most regulated of which are bFGF, Adiponectine, ENA78, GRO, GRO-a, and ICAM1-3. Also, ADRC-conditioned media that are created under hypoxic conditions promote HUVECs to express pro-angiogenic factors. Garcia et al. concluded that an enhanced angiogenic ability of ADRCs after hypoxia preconditioning exists [115]. In addition, Su et al. demonstrated that diabetic microenvironment preconditioning of ADRCs can increase the secretion of cytokines related to M2 macrophage polarization (IL-6, MCP-1, etc.). Furthermore, the treatment of those preconditioned ADRCs effectively maintained blood glucose homeostasis, reduced insulin resistance, promoted islet regeneration, and improved diabetic complications in rats (chronic kidney disease, non-alcoholic steatohepatitis, lung fibrosis, and cataracts) [116]. Moreover, He et al. demonstrated that mechanical stretch-preconditioned ADRCs have improved survival and immunoregulatory characteristics in vitro. When those preconditioned ADRCs were transplanted into chronic wounds, the wound region spotted considerably faster closure and reduced levels of inflammatory mediators, owing mostly to macrophage polarization induced by the transplanted ADRCs [117].

### 5.4. Biomaterial Approaches

In recent years, the merging of stem cell therapy with biomaterial approaches has resulted in the development of numerous promising techniques for tissue and organ regeneration. Yang et al. [118] discovered that ADRC-Exos encapsulated in PF-127 hydrogel can be taken up by HUVECs and improves their migration, proliferation, and tubule formation in vitro. Their effects on promoting angiogenesis were mediated by the HIF-1α/VEGF signaling pathway, suggesting that ADRC-Exos encapsulated in PF-127 hydrogel play a crucial role in regulating angiogenesis and offer a promising treatment option for autologous fat grafting. Kim et al. [119] investigated that spheroid-type three-dimensional cell masses (3DCM) composed of ADRCs that were cultured on a substrate with immobilized fibroblast growth factor 2 included vascular cells (CD34+/CD31+/KDR+/α-SMA+) with high production of VEGF, and infarct size and cardiomyocyte apoptosis were reduced in the 3DCM-injected group compared with the ADRC-injected group. In addition, numerous transplanted cells developed into smooth muscle and endothelial cells, creating vascular networks that were integrated into the host arteries. Moreover, we investigated the angiogenic and tissue-preserving properties of mesenchymal stem cell sheets produced with a new magnetic tissue engineering technique, and these effects were linked to decreased apoptosis in ischemic tissues and enhanced production of VEGF [120]. Additionally, we found that ADRC sheets created with the Mag-TE method implantation also promote angiogenesis in the border zone and infarct area in WT mice following myocardial infarction, and the angiogenic effects of ADRC sheets are linked to elevated VEGF and bFGF mRNA expression in ischemic hearts [81]. These results could be useful in the development of tissue engineering methods.

## 6. Challenges and Future Perspectives in ADRC for Therapeutic Angiogenesis

Due to their accessibility, ease of isolation in large quantities, and capacity to differentiate into multiple cell lineages, ADRCs continue to be a very promising solution in regenerative medicine. However, there are still some issues that need to be resolved before their angiogenetic potential can be applied to clinical practice. As discussed in the main text, ADRCs are not a single type of cell but rather a heterogeneous population with varying characteristics depending on their sources and surface markers. It has been reported that their properties differ accordingly. A key challenge moving forward is to scientifically verify which characteristics of ADRCs from which sources can maximize both safety and efficacy. From a quality control perspective, it is also necessary to clarify the differences between fresh ADRCs and cryopreserved ADRCs. Furthermore, in clinical practice, discussions are needed regarding the appropriate administration method—whether systemic administration via intravenous injection is sufficient or if direct transplantation into target organs is required. These considerations should take both therapeutic efficacy and safety into account. To keep consistency in the therapeutic potential of ADRCs, it is essential to standardize the protocol for ADRC isolation and expansion. Additionally, the effectiveness and safety of the materials utilized to isolate, culture, and preserve ADRCs need to be investigated. In addition, the mechanisms behind ADRC differentiation and immunomodulation are not well understood. Therefore, future research needs to concentrate on the characteristics and functional mechanisms of ADRCs. Also, with the development of bioengineering and gene editing, it is feasible to optimize the differentiation and integration of ADRCs into the host. In general, there are still several challenges remaining in ADRC application, and the combination of other treatments could be one of the solutions to enhance effectiveness. Moreover, advancements in nanobiotechnology and genetic engineering technologies could hold promise for achieving better outcomes. Most importantly, while the initial effects of this treatment have been favorable, long-term adverse events and complications remain unknown at this stage. Therefore, continuous monitoring of patients who have undergone ADRC therapy is essential. Additionally, in therapeutic angiogenesis using bone marrow-derived cells, we reported that disease-specific responses have been observed in the mid-to-long term [121,122,123]. For example, in cases of critical limb ischemia, it has been reported that patients with peripheral artery disease (caused by atherosclerosis) show a weaker therapeutic response compared to those with Buerger’s disease [121]. Thus, the long-term outcomes of this therapy should also be evaluated.

## 7. Conclusions

ADRCs show remarkable therapeutic potential in angiogenesis ability, and their easy accessibility from adipose tissue and characteristics make them a promising option for regenerative medicine. More research is attempting to evaluate the safety and effectiveness as well as the protective mechanisms of ADRCs because of the increasing evidence of ADRC therapy in improving ischemic diseases. Although some of the direct and indirect mechanisms of ADRCs have been addressed, further research is still needed to fully clarify the protective mechanisms of ADRCs. In order to utilize the full regenerative potential of ADRCs for tissue repair and regeneration, future investigations need to target those mechanisms and translate them into further clinical applications in treating ischemic diseases.

## Figures and Tables

**Table 1:** Clinical trials of ADRC therapy in ischemic diseases.

Diseases	Number of Patients	Intervention		Follow-Up	Outcomes	Trail ID/Ref	Year
		Route of Administration	Doses				
Ischemic stroke	20	Intravenous	1 × 10^6^ cells/kg	24 months	Safe and well tolerated; no infusion reactions; improvement in the NIHSS scores	NCT01678534 [35]	2018
Ischemic stroke	3	Intracerebral	1 × 10^8^ cells	6 months	No related safety issue; improvements in neurological functions	NCT02813512 [36]	2018
Stroke	400	Intravenous	1 × 10^6^ cells/kg	6 months	Angiogenic effect	NCT02849613	2021
Ischemic stroke	30	Intravenous	1 × 10^6^ cells/kg	24 months	Ongoing	NCT04280003 [37]	2023
Myocardial ischemia	45	Intramyocardial	0.4 × 10^6^ cells/kg	12 months	Terminated on 2014 due to delay	NCT01556022	2016
Herat failure	10	Intramyocardial	100 × 10^6^ cells	6 months	Improvement of cardiac function	NCT02387723 [38]	2016
Ischemic heart disease	133	Intramyocardial	100 × 10^6^ cells	6 months	Improvement in New York Heart Association (NYHA) class	NCT02673164 [39]	2020
Heart failure	81	Intramyocardial	100 × 10^6^ cells	6 months	Safety; improvement of quality-of-life	NCT03092284 [40]	2022
Critical limb ischemia	13	Intramuscular	100 × 10^6^ cells	6 months	Safety; improvement of wound healing	NCT01211028 [41]	2017
Critical limb ischemia	33	Intra-arterial	0.5 × 10^6^ cells/kg 1 × 10^6^/kg	12 months	Completed; no results posted	NCT01745744 [42]	2018
Critical limb ischemia	20	Intramuscular	(100–300) × 10^6^ cells	12 months	Improvement of blood flow	NCT01663376 [43]	2019
Critical limb ischemia	5	Intramuscular	0.13–6.4 × 10^7^ cells	6 months	Safety; angiogenesis effect; suppression of tissue inflammation	UMIN000010143 [44]	2020
Critical limb ischemia	29	Intramuscular	0.11–13.5 × 10^7^ cells	6 months	No major adverse event; optimal survival rate; improvement of numerical rating scale as QOL score, ulcer size, and 6-min walking distance	jRCTb040190118 [45]	2022
Critical limb ischemia	20	Intramuscular	1 × 10^7^ cells/1 mL/vial 1 × 10^8^ cells/1 mL/vial	6 months	Ongoing	NCT04661644 [46]	2024
Chronic kidney disease	39	Intravenous	6.4 × 10^7^ cells 19.2 × 10^7^ cells 32.0 × 10^7^ cells	12 months	Well tolerated	NCT02933827 [47]	2024
Chronic kidney disease	10	Intravenous	75 × 10^6^ cells 150 × 10^6^ cells	22 months	Ongoing	NCT04869761	2024

## Data Availability

No new data were generated.
